# Production of *para*-aminobenzoic acid from different carbon-sources in engineered *Saccharomyces cerevisiae*

**DOI:** 10.1186/s12934-016-0485-8

**Published:** 2016-05-26

**Authors:** Nils J. H. Averesch, Gal Winter, Jens O. Krömer

**Affiliations:** Centre for Microbial Electrochemical Systems (CEMES), The University of Queensland, Office 618, Level 6 Gehrmann Building (60), St. Lucia, Brisbane, QLD 4072 Australia; Advanced Water Management Centre (AWMC), The University of Queensland, Brisbane, Australia; School of Science and Technology, University of New England, Armidale, Australia

**Keywords:** pABA, Phenylethanol, Aromatics, Yeast, Glycerol, Ethanol

## Abstract

**Background:**

Biological production of the aromatic compound *para*-aminobenzoic acid (pABA) is of great interest to the chemical industry. Besides its application in pharmacy and as crosslinking agent for resins and dyes pABA is a potential precursor for the high-volume aromatic feedstocks terephthalic acid and *para*-phenylenediamine. The yeast *Saccharomyces cerevisiae* synthesises pABA in the shikimate pathway: Outgoing from the central shikimate pathway intermediate chorismate, pABA is formed in two enzyme-catalysed steps, encoded by the genes *ABZ1* and *ABZ2*. In this study *S. cerevisiae* metabolism was genetically engineered for the overproduction of pABA. Using in silico metabolic modelling an observed impact of carbon-source on product yield was investigated and exploited to optimize production.

**Results:**

A strain that incorporated the feedback resistant *ARO4*^*K229L*^ and deletions in the *ARO7* and *TRP3* genes, in order to channel flux to chorismate, was used to screen different *ABZ1* and *ABZ2* genes for pABA production. In glucose based shake-flaks fermentations the highest titer (600 µM) was reached when over-expressing the *ABZ1* and *ABZ2* genes from the wine yeast strains AWRI1631 and QA23, respectively. *In silico* metabolic modelling indicated a metabolic advantage for pABA production on glycerol and combined glycerol-ethanol carbon-sources. This was confirmed experimentally, the empirical ideal glycerol to ethanol uptake ratios of 1:2–2:1 correlated with the model. A ^13^C tracer experiment determined that up to 32 % of the produced pABA originated from glycerol. Finally, in fed-batch bioreactor experiments pABA titers of 1.57 mM (215 mg/L) and carbon yields of 2.64 % could be achieved.

**Conclusion:**

In this study a combination of genetic engineering and in silico modelling has proven to be a complete and advantageous approach to increase pABA production. Especially the enzymes that catalyse the last two steps towards product formation appeared to be crucial to direct flux to pABA. A stoichiometric model for carbon-utilization proved useful to design carbon-source composition, leading to increased pABA production. The reported pABA concentrations and yields are, to date, the highest in *S. cerevisiae* and the second highest in a microbial production system, underlining the great potential of yeast as a cell factory for renewable aromatic feedstocks.

**Electronic supplementary material:**

The online version of this article (doi:10.1186/s12934-016-0485-8) contains supplementary material, which is available to authorized users.

## Background

### Significance of bio-derived aromatic feedstocks for chemical industry

Microbial metabolic pathways give rise to many compounds that can potentially substitute currently petroleum based chemicals with bio-derived ones or replace them with bio-based alternatives. Using metabolic engineering these capabilities are already being exploited to create new organisms that use abundant and renewable feedstocks to efficiently produce an expanding spectrum of valuable chemicals [1–3]. Recent advances in metabolic engineering of *Saccharomyces cerevisiae* have shown that systems and synthetic biology tools nowadays allow rational construction of capable production strains [4–6]. In particular aromatics have great potential for bio-based production as the shikimate pathway gives rise to a wealth of aromatics and derived compounds, with diverse applications in different industries, including the chemical one [7–12].

The shikimate pathway intermediate *para*-aminobenzoic acid (pABA) is one of these aromatics with versatile applicability, it is already being used use as crosslinking agent for resins and dyes, as a precursor in the pharmaceutical industry and as a therapeutic itself (e.g. for the drug POTABA^®^). However it has so far not been applied as bulk feedstock for large scale application in the plastic and fibre industry, likely because it is not price-competitive compared to other precursors that are readily available from chemical synthesis. This may change once a competitive bio-based production has been established: It has recently been demonstrated that pABA can be chemically converted to terephthalic acid [13] making it a potential bio-feedstock for polyethylene terephthalate (PET) production. PET is used for packaging as well as clothing and recently also in the auto industry [14]. The global market volume for PET packaging was nearing 16 Mt valued at 48.1 billion USD in 2014 [15]. This is expected to rise to 19.9 Mt and 60 billion USD in 2019 [15]. The global market volume for bio-based PET exceeded 540 kt in 2012 and is expected to rise sharply till 2020, with a doubling in 2015 and a tripling in 2016 in respect to 2014. While current bio-based PET is claimed to be up to 30 % sustainable [16] in fact only the monoethylene glycol share of the copolymer is derived from renewable feedstocks, while the terephthalic acid component, the major part of PET by volume and cost, is still petroleum based. Therefore industrial interest to develop a fully sustainable PET product is high and substantial investments are being made in order to establish bio-based terephthalic acid production [14, 17, 18].

pABA may also be chemically converted to *para*-phenylenediamine (e.g. via Kochi- or Hunsdiecker reaction followed by nucleophilic substitution), which is (besides terephthalic acid) the second building block of the aramid fibre Kevlar^®^. Global demand for these materials, which combine high strength with low weight as well as chemical and heat resistance properties, was estimated to 74.5 kt in 2014 and is expected to rise to 110 kt in 2020 with an estimated value of 4.7 billion USD [19].

### Aromatic compounds derived from yeast shikimate pathway

**Fig. 1:**
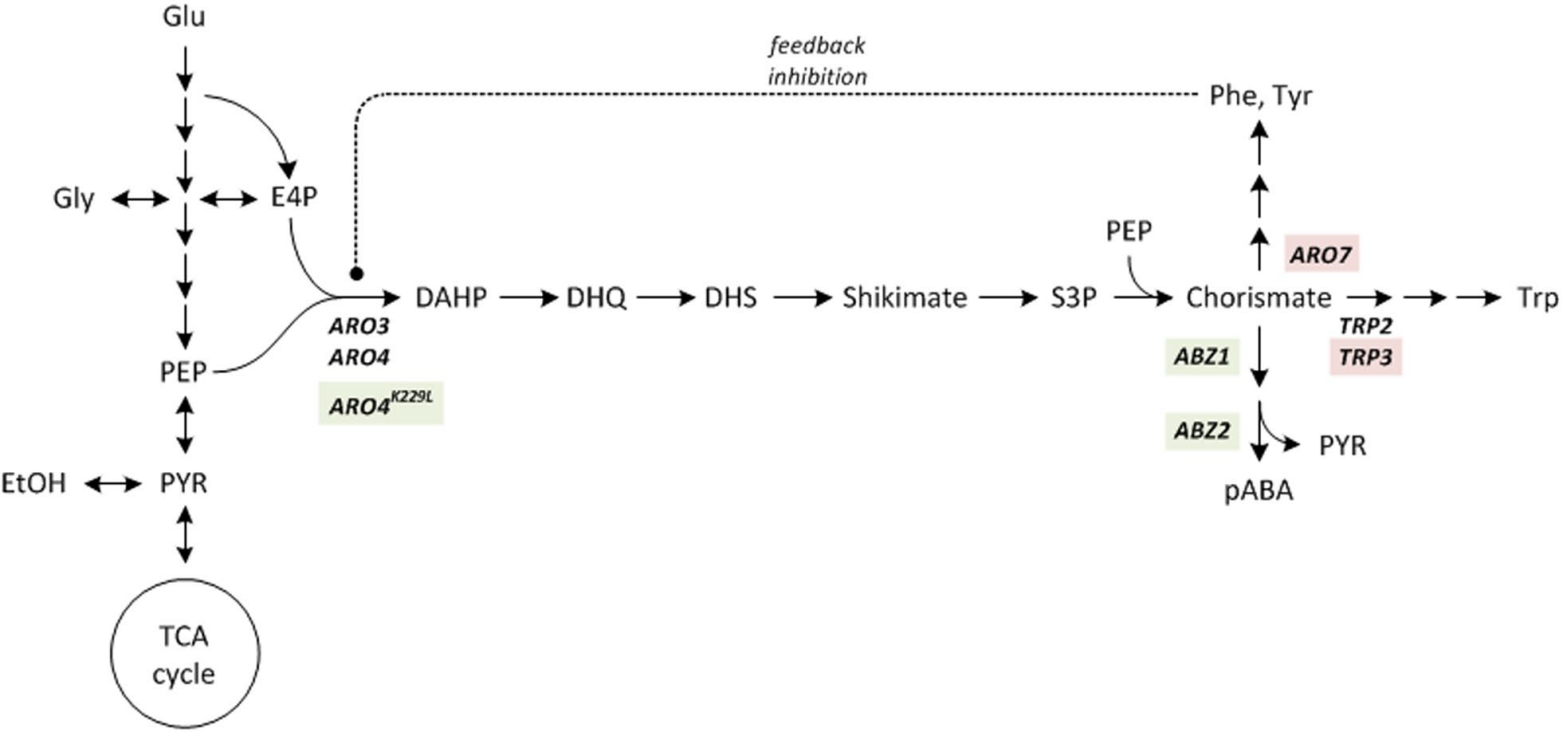
Simplified shikimate pathway including modifications for pABA production. Knock-out targets are highlighted* red*, (over)expression targets* green*. The central intermediates are: 3-deoxy-d-arabino-heptulosonate-7-phosphate (DAHP), 3-dehydroquinate (DHQ), 3-dehydroshikimate (DHS), shikimate, shikimate-3-phosphate (S3P), chorismate, phenylalanine (PHE), tyrosine (TYR), tryptophan (TRP) and *para*-aminobenzoate (pABA). Important genes and the respective enzymes involved are: *ARO3*/*ARO4*: 3-deoxy-d-arabino-heptulosonate-7-phosphate (DAHP) synthase, *ARO7*: chorismate mutase, *TRP2*: anthranilate synthase, *TRP3*: indole-3-glycerol-phosphate synthase, *ABZ1*: aminodeoxychorismate synthase, *ABZ2*: aminodeoxychorismate lyase

pABA is naturally produced by yeast as an intermediate in the shikimate pathway, the central metabolic route leading to formation of the aromatic amino acids phenylalanine, tyrosine and tryptophan [20]. The pathway is simplistically reproduced in Fig. 1: The intermediates of glycolysis and pentose phosphate pathway, phosphoenol pyruvate (PEP) and erythrose 4-phosphate (E4P), respectively, enter the pathway through a series of condensation and redox reactions that yield the intermediate shikimate. Shikimate is subsequently converted to the central branch point metabolite chorismate under ATP hydrolysis and introduction of a second PEP. Outgoing from chorismate the aromatic amino acids and pABA are synthesised, the latter being a precursor for folate metabolism [21]. The synthesis of pABA comprises two key enzymes encoded by the genes *ABZ1* and *ABZ2*: The gene product of *ABZ1* (aminodeoxychorismate synthase) transfers an amino-group from glutamine to the *para*-position of chorismate, thus forming glutamate and 4-amino-4-deoxychorismate. The gene product of *ABZ2* (aminodeoxychorismate lyase) then cleaves the ester-bond between the ring and the C3-body releasing pyruvate and pABA.

Regulation of the shikimate pathway in *S. cerevisiae* is manifold [22] and yet to be fully understood. It is tightly controlled by its end-products, foremost by inhibition of its initial step by phenylalanine and tyrosine [23]. However a point mutation in the *ARO4* gene (leading to a changed amino acid sequence, *ARO4*^*K229L*^) has been reported that makes the gene product largely resistant to feedback inhibition [23].

Despite the biosynthesis route to pABA being present in *S. cerevisiae*, common laboratory strains like S288c and CEN.PK derived ones, suffer a growth deficit if pABA is omitted from media, supposedly due to reduced functionality of the *ABZ1* gene [24]. In the comparative study that examined this, it was also found that the wine yeast EC1118 carries a more functional version of this gene [24]. In fact, a previous study on pABA production in S288c showed that overproduction could be achieved using the *ABZ1* gene from the wine yeast AWRI1631, which is identical to the one of EC1118 [9]. The study identified yeast as suitable host, with regard to in silico determined theoretical maximum yields (0.53 g/g_Glucose_) and in vivo toxicity limits (50 % growth rate reduction at 0.62 g/L) [9]. However, the production was moderate (250 µM), raising the question, if *ABZ2* might be currently limiting pABA production in yeast. Recently overproduction was reported in *E. coli* (4.8 g/L) where also the choice of the bacterial analogues to *ABZ1* and *ABZ2* (*pabA* & *pabB*/*pabAB* and *pabC*) was shown to be crucial [25].

In the present study overproduction of pABA in *S. cerevisiae* from different carbon-sources is improved using genetic engineering and metabolic modelling. In particular the metabolic bottleneck to pABA formation from chorismate and the use of glycerol as an alternative carbon-source were investigated. Glycerol is a by-product of both the biodiesel and the bioethanol industries and is considered an inexpensive and sustainable feedstock that does not directly compete with resources of food industry [26, 27]. In this study a mixed glycerol-ethanol (GLY/ETH) feed with a high glycerol-share was used to maximize pABA production and yield.

## Results and discussion

### Designing a strain for pABA production—screening of different *ABZ1* and *ABZ2* genes

The production of pABA in *S. cerevisiae* requires diversion of carbon flux towards pABA synthesis through elimination of competing metabolic pathways and enhancement of pABA synthesis through over-expression of key enzymes. Therefore the *TRP3* and *ARO7* genes were deleted and the feedback inhibition resistant *ARO4*^*K229L*^ gene was introduced, in order to overcome product inhibition and increase flux to the shikimate pathway [8, 10, 12].

**Fig. 2:**
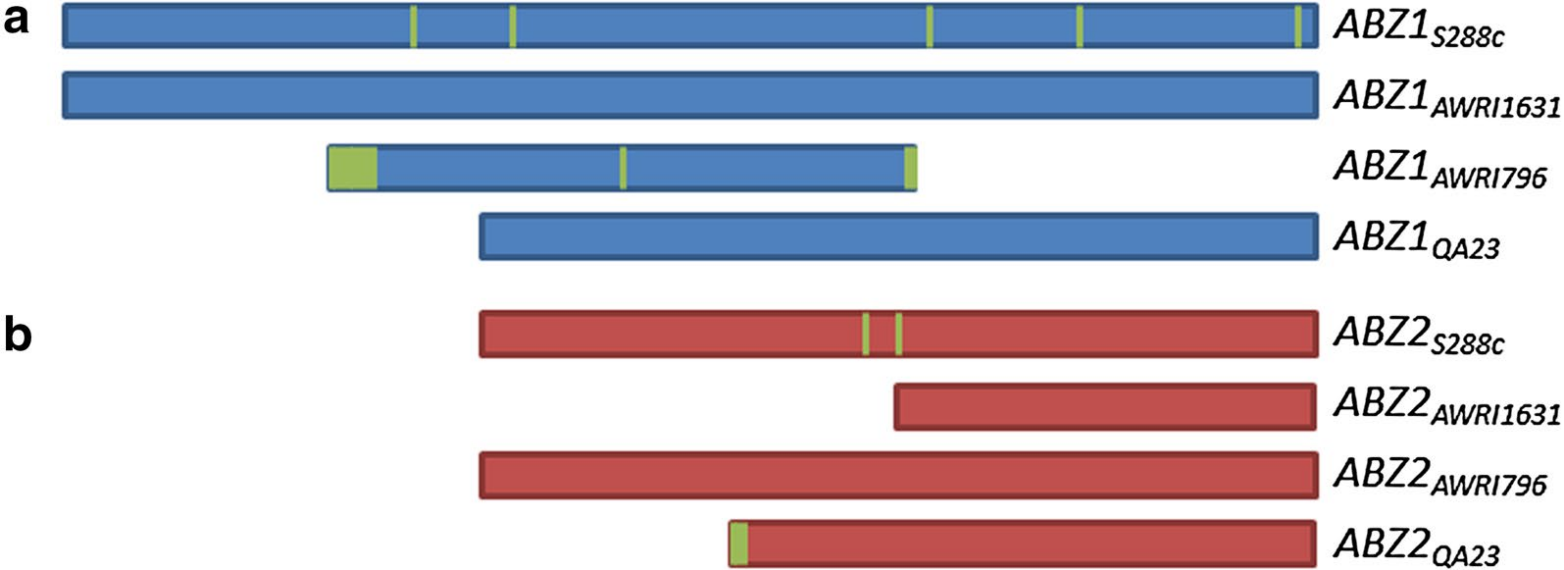
Simplified depiction of the alignment of *ABZ1* (**a**) and *ABZ2* (**b**) amino acid sequences. Amino acid residues unique to a certain sequence are indicated in *green*. Sequences are shown from N-terminus to C-terminus

The resulting genetically modified base strain “PABA0” (cf. Fig. 1; “Strain and plasmid construction” section) was used as a chassis cell to screen different alleles of *ABZ1* and *ABZ2* for their effect on pABA production. To start with, sequences were compared in silico, by means of the alignment of the 43 different *ABZ1* and *ABZ2* nucleotide and amino acid sequences, derived from SGD (www.yeastgenome.org). With the aim to cover the greatest possible variety, the alleles of the lab yeast (S288c) and three wine yeasts (AWRI1631, AWRI796, QA23) were chosen. These *ABZ1* and *ABZ2* genes were compared in detail regarding sequence similarities and differences (Fig. 2; cf. Additional file 1 for full sequence analysis).

The *ABZ1* genes (Fig. 2a) distinguished not only by SNPs, but also appeared in different ORFs. So did the *ABZ2* genes, furthermore it was found that the region upstream of the annotated ORF of *ABZ2*_*AWRI1631*_ coincides with the nucleotide sequence present on the genome of AWRI796. This means that the full ORF of *ABZ2*_*AWRI796*_ is present also in AWRI1631. This may indicate a false annotation of either gene. Therefore both possible ORFs (cf. Fig. 2b) were included for screening, the putatively falsely annotated gene is referred to as *ABZ2*_*AWRI1631*_½ (which corresponds to the original annotation of *ABZ2*_*AWRI1631*_) the other one as *ABZ2*_*AWRI1631*_full (which corresponds to the original annotation of *ABZ2*_*AWRI796*_). The yeast strain Lalvin QA23 provided other substantially different *ABZ1* and *ABZ2* genes. All four *ABZ2* genes and the *ABZ1* genes of AWRI1631 and QA23 were chosen for in vivo comparison.

**Fig. 3:**
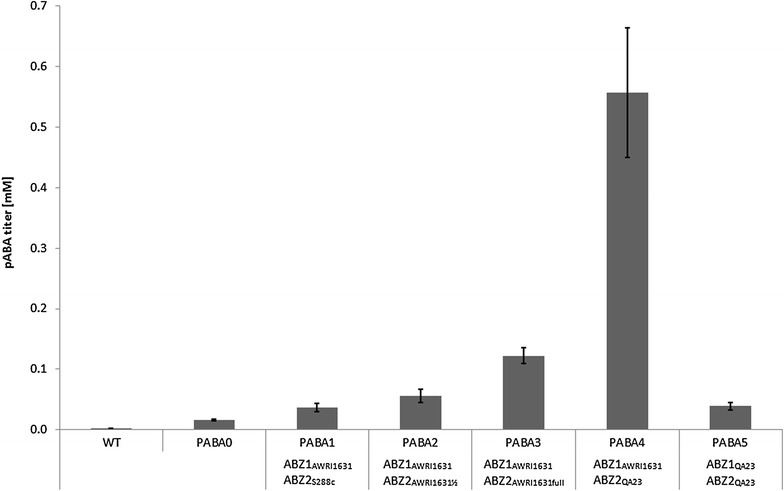
Production of pABA in shake flask experiments of *S. cerevisiae* strains. WT = CEN.PK113-5D, PABA0 = CEN.PK113-5D Δ*trp3* Δ*aro7* *ARO4* ^*K229L*^. PABA1–PABA5 are based on PABA0, with over-expression of additional *ABZ1* and *ABZ2* alleles on a plasmid expression vector as indicated on the figure (for full genotype cf. “Strain and plasmid construction” section)

The strains “PABA1”—“PABA5” (cf. “Strain and plasmid construction” section), which over-expressed the different *ABZ1* & *ABZ2* genes, were compared in shake-flask experiments with the aim to find the best set of *ABZ1* and *ABZ2* genes for high pABA production (Fig. 3). PABA0 already exhibited baseline pABA production in respect to the background strain (CEN.PK113-5D), indicating that the modifications so far already directed flux towards pABA, even without increased expression of *ABZ1* and *ABZ2*. This was supported by the observation that the strain grew without supplementary pABA (cf. “In vivo restrictions of carbon-source utilization and impact on aromatic product spectrum” section). Over-expression of *ABZ1* and *ABZ2* led in all cases to pABA production higher than from the base strain PABA0. The highest production was achieved by the combination of *ABZ1*_*AWRI1631*_ and *ABZ2*_*QA23*_, which showed a ~35-fold increase in the pABA concentration compared to PABA0. Further it can be noted that the full *ABZ2*_*AWRI1631*_ gene in PABA3 led to a higher production than the truncated version expressed in strain PABA2. Finally, PABA5, harbouring *ABZ1*_*QA23*_ in combination with *ABZ2*_*QA23*_, was inferior compared to PABA2, PABA3 and PABA4.

It is evident that a connection between pABA production and gene sequences exists. Inferred from the marginal pABA production of strain PABA1 it appears that not only the *ABZ1* but also the *ABZ2* gene of S288c and *S. cerevisiae* strains with an identical sequence has reduced functionality. The most obvious explanation seems to be that the two differences in the amino acid sequence of *ABZ2*_*S288c*_ in respect to *ABZ2*_*AWRI796*_ (cf. Fig. 2) reduce the functionality of the enzyme. With PABA3 producing nearly twice the amount of pABA compared to PABA2 it is evident that *ABZ2*_*AWRI1631*_ was annotated falsely in the AWRI1631 genome, and the larger ORF (corresponding to *ABZ2*_*AWRI796*_) is the correct one. When including PABA4 in the comparison *ABZ2*_*QA23*_ appears to excel in pABA production. Comparing its sequence to the other genes it seems very likely that a relation between the unique N-terminus of *ABZ2*_*QA23*_ and its effect on pABA production exists. Comparing PABA4 to PABA5 shows that also the choice of *ABZ1* has a great influence, as only combining *ABZ1*_*AWRI1631*_ with *ABZ2*_*QA23*_ allowed greatly increased pABA titers. It seems likely that *ABZ1*_*QA23*_ is dysfunctional due to the 5′-end truncation of the gene.

The differences that arise for pABA production may not be only due to differences in activity of these enzymes but also enzyme concentration. This may involve more complex regulation on transcriptional and translational levels like e.g. codon usage or mRNA stability [28]. Therefore expression levels were examined by measuring the level of target gene mRNA using RT-qPCR. mRNA levels of the different *ABZ1* and *ABZ2* genes in the production strains were compared to the background strain with no *ABZ1* and *ABZ2* over-expression (Table 1). In all over-expressing strains significantly increased mRNA levels of the ABZ genes were found, which correlates with the observed higher pABA production. By relating the abundance of transcript of the different genes to pABA production in the different strains, conclusions towards their activity can be drawn. It can be hypothesized that *ABZ1*_*AWRI1631*_ codes for a more active enzyme than *ABZ1*_*QA23*_, as mRNA levels of *ABZ1*_*AWRI1631*_ are significantly lower, but the gene leads to significantly higher pABA production. Further the putatively high expression of *ABZ2*_*S288c*_ and *ABZ2*_*AWRI1631*_½ but low pABA production in strains carrying these genes indicate low activity. In reverse this indicates higher activity of *ABZ2*_*AWRI1631*_full as it leads to higher pABA production although its transcript is least abundant. The obtained maximum pABA production from *ABZ2*_*QA23*_ may however be due to its higher expression. Therefore, the codon usage of the different ABZ genes was compared (cf. Additional file 1) in order to validate the relevance of mRNA levels for assessment of gene expression. However no major differences could be found that would indicate greatly different strength of translation of the ABZ transcripts.

**Table 1:** Relative mRNA levels of the different over-expressed ABZ genes during exponential growth on glucose

Gene/origin	Background	S288c	AWRI1631	QA23
*ABZ1*	0.2 ± 0.0	NA	12 ± 3	60 ± 12
*ABZ2*	0.5 ± 0.1	175 ± 44	201 ± 28^a^/16 ± 4^b^	119 ± 0

*NA* not applicable

^a^ *ABZ2*_AWRI1631_½

^b^ *ABZ2*_AWRI1631_full

Finally, relating ligand binding sites to the mutations in amino acid sequences of the different ABZ genes could allow conclusions towards enzyme functionality to be drawn (Additional file 1) and may explain the greatly improved performance of certain genes.

### *In silico* analysis of carbon-source composition leads to enhanced pABA production in vivo

During characterization experiments of the PABA strains it could be observed that the production of pABA was higher later in the fermentation, when glucose was depleted and glycerol & ethanol were reassimilated (cf. “In vivo restrictions of carbon-source utilization and impact on aromatic product spectrum” section). To understand this phenotype in silico modelling was used: Elementary mode analysis was performed [29], in order to compare biomass and product carbon-yields on the substrates glucose, glycerol, ethanol and glycerol + ethanol. The achievable maximum carbon yield for pABA from ethanol is generally lower than from glucose or glycerol, while glycerol allows a higher theoretical pABA yield than glucose (Table 2; cf. Additional file 2 for distribution of elementary flux modes). The highest possible carbon yield for pABA with and without biomass formation, can be achieved in silico from a combination of glycerol and ethanol as feed, which may explain our experimental observation of high pABA formation in the second fermentation phase when reutilization of ethanol and/or glycerol occurs.

**Table 2:** Theoretical maximum product carbon yields using different substrates

Substrate	Glucose	Ethanol	Glycerol	Glycerol + ethanol
Maximum pABA yield (%)	84	75	90	92
Maximum pABA yield with biomass (%)	81	50	86	87

**Fig. 4:**
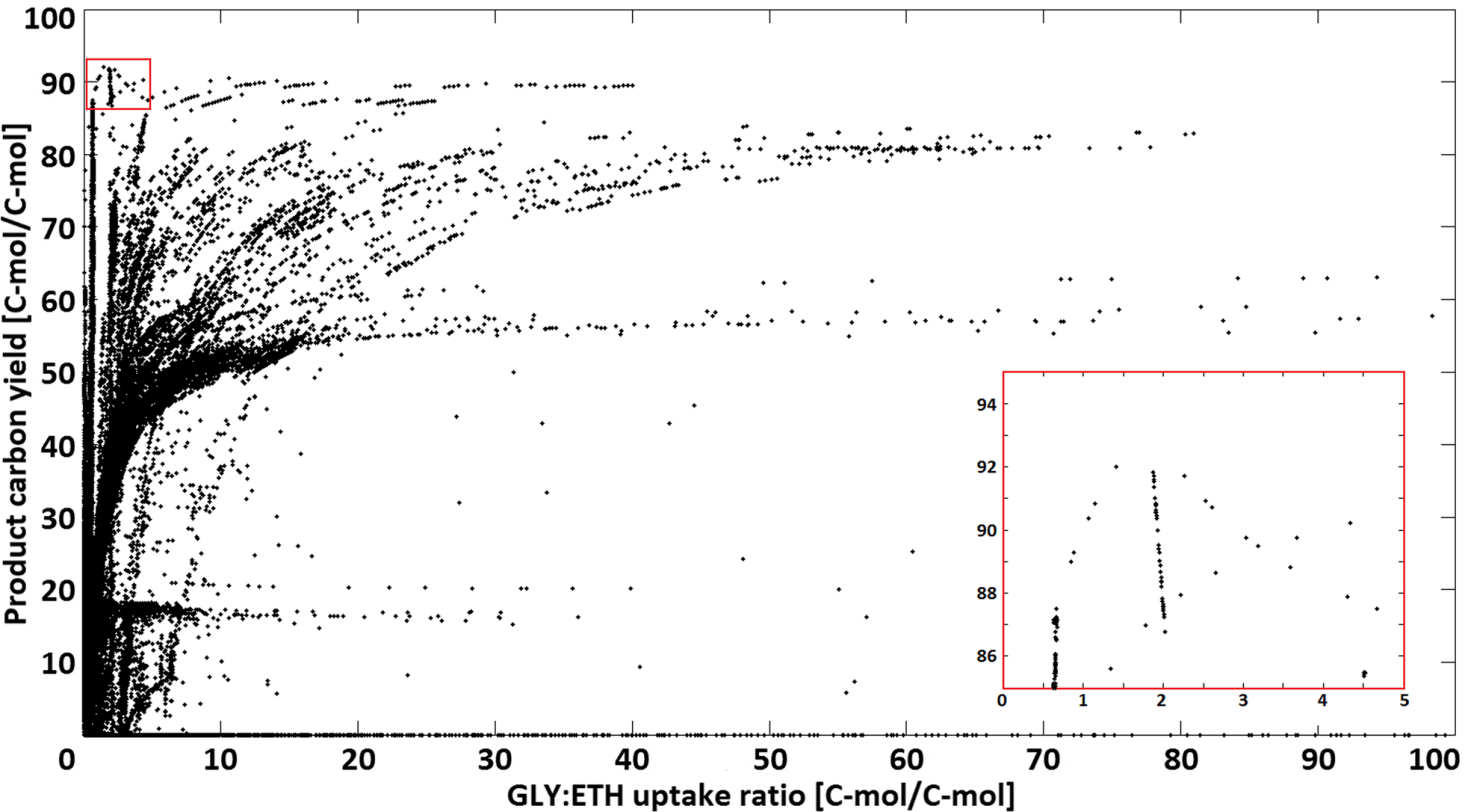
Distribution of pABA yields in dependence of the glycerol to ethanol ratio in elementary flux modes in a window with a GLY:ETH ratio ≤100 [C-mol/C-mol]. Each point in the chart corresponds to the specific product carbon yields [%] and substrate ratio of the respective elementary flux mode. The insert at the *bottom right corner* is a magnification of the area in the *top left corner* where the modes with the highest yields are located

The analysis of the product carbon yields from all elementary modes on GLY/ETH-feed allowed the determination of an ideal ratio between glycerol and ethanol. Figure 4 shows that the majority of feasible flux distributions feature a GLY:ETH ratio lower than 10, while the highest product yields are obtained with GLY:ETH ratios between 0.5 and 2 (magnified area in Fig. 4).

Increased maximum yield using glycerol as carbon-source (compared to glucose) can be explained with additional reducing power (FADH_2_) gained during glycerol metabolization (reaction R11b, Additional file 2), which can be used to regenerate ATP. The initial step of ethanol utilization (reaction R10c, Additional file 2) also reduces an extra redox co-factor (NADH); however on ethanol alone very limited metabolic freedom exists, as all flux to upstream pathways (glycolysis, pentosephosphate pathway, shikimate pathway) has to be derived from acetyl-CoA via phosphoenolpyruvate carboxykinase (reaction R41c, Additional file 2) and malic enzyme (reaction R62c, Additional file 2). In particular for formation of the shikimate pathway precursor PEP from ethanol, two more mol ATP are required in respect to glucose and four more in respect to glycerol.

This is compensated on a combined GLY/ETH-feed: Separate carbon flow to the upper and lower parts of central metabolism allows full advantage of higher availability of reduced redox equivalents, resulting in a higher biomass yield and more modes close to the stoichiometric limit (line between max. biomass and max. product yield, cf. Additional file 2 for biomass vs. product yield plots). In addition the majority of modes with low product/biomass formation are abolished, as the two major by-products on glucose (GLY/ETH) are substrates now.

**Fig. 5:**
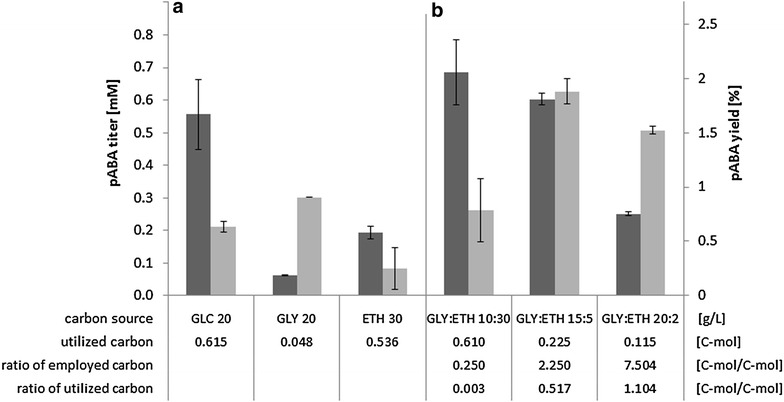
pABA titers (*dark grey*) and yields (*light grey*) achieved by PABA4 on different carbon-sources (**a**) and GLY:ETH different ratios (**b**). Titers are evaporation corrected and represent the maximum after either carbon-source was used up and/or no further product formation occurred. Utilized carbon is the amount of C-mol from either carbon-source that was taken up and actually metabolised. Yields and carbon usage ratios are based on the evaporation corrected titers while only respecting the actually metabolized fraction of the carbon-source

In order to reproduce the observed higher pABA production of glucose batches after the diauxic shift (when re-utilizing glycerol and/or ethanol) and to test the prediction of the elementary flux mode analysis, the impact of the carbon-sources glucose, glycerol, ethanol and glycerol + ethanol mixtures was tested in vivo using the PABA4 strain in shake-flask experiments. Comparing single carbon-sources the pABA titer was found to be highest on glucose, the carbon yield however was higher on glycerol (Fig. 5a). The product carbon yield from ethanol can be noted as lowest, despite a high uncertainty due to high evaporation (cf. “Metabolite analysis” section). The highest pABA yields and also titers were obtained when combining glycerol and ethanol. Therefore different concentrations and ratios of glycerol and ethanol were tested to determine the ideal ratio of glycerol to ethanol (Fig. 5b). It was found that GLY:ETH ratios in the feed >3:1 g/g allowed for uptake ratios >0.5 C-mol/C-mol, delivering the highest pABA yields. Enforcing an increase in uptake ratio only worked up to a certain degree: A 20:2 g/g GLY:ETH feed-ratio only allowed an uptake ratio of 1.1 C-mol/C-mol and while pABA yield was only slightly reduced, titer was more than halved.

The results indicate that both, GLY and ETH, are utilized and contribute to pABA production, however in different ways. Therefore the contribution of the carbon from the two substrates to pABA formation on a combined GLY/ETH carbon-source was investigated in a tracer experiment with 1,2-^13^C labelled ETH and natural GLY. Enrichment analysis of pABA revealed the share of each substrate’s contribution: In total up to 31 % of pABA were derived from GLY (mean enrichment = 69 %). Further analysis of the labelling of amino acids revealed that glycerol was mainly metabolized in the upper part of glycolysis and pentose phosphate pathway, with little to no C^12^-carbon reaching the TCA-cycle (mean enrichment of threonine, aspartate and glutamate 92.2, 92.9, 93.9 %, respectively). The activity of the threonine aldolase pathway for glycine biosynthesis could be seen by a glycine enrichment of 79.5 %, while serine was only labelled 59.8 % and clearly was also derived from glycerol. Since the alanine enrichment reached 85.2 % it seems that glycerol did not reach the pyruvate pool to a large extent. Ethanol finally also reached the pentose phosphate pathway and subsequently the biosynthesis of histidine (44.1 % enriched) and the aromatics. The fact that ethanol served as a precursor for pentose phosphate pathway derived compounds points towards a limitation in the supply of C_3_ molecules from the glycerol uptake. During the tracer experiment the GLY:ETH uptake ratio was 1.1 C-mol/C-mol. It is striking that the contribution of ethanol to amino acid biosynthesis as well as pABA formation (GLY:ETH ratio of 0.47 C-mol/C-mol) was greater than the uptake ratio. This means that gluconeogenesis from ethanol was active while glycerol was metabolised. This could point towards metabolic channelling [30]. Glycerol could for instance contribute to a larger extend to synthesis of carbohydrates.

### In vivo restrictions of carbon-source utilization and impact on aromatic product spectrum

**Fig. 6:**
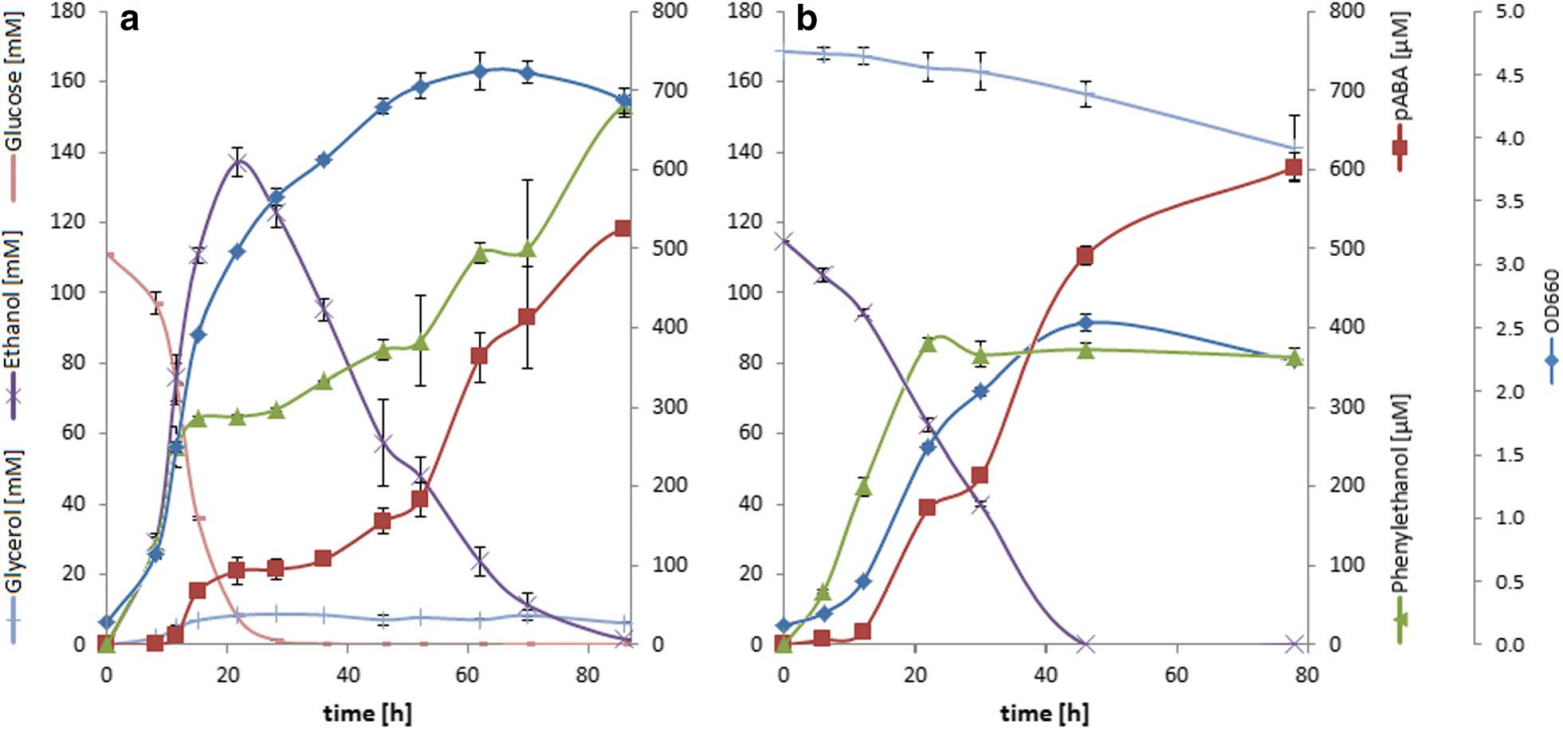
Time course of substrate consumption and product formation of biomass main aromatic products on GLC (**a**) and GLY/ETH (**b**). Titers are corrected for evaporation of H_2_O to reflect comparable metabolite concentrations. Carbon sources were 20 g/L glucose (initial 0.666 C-mol, of which 0.615 were consumed) in (**a**) and 15 g/L ethanol + 5 g/L glycerol (initial 0.722 C-mol, of which 0.225 were consumed) in (**b**). Biomass is reflected by means of the OD at 660 nm. Growth rates in the exponential phase were µ = 0.2242 h^−1^ on glucose and µ = 0.1081 h^−1^ on glycerol/ethanol

To characterize the higher in vivo pABA production on GLY/ETH in more detail, the metabolic profiles over the course of fermentations on GLC and GLY/ETH were compared (Fig. 6): On GLC the maximum pABA titer was reached only after almost 130 h while a major part of pABA was produced during re-assimilation of ethanol/glycerol. On GLY/ETH the maximum pABA titer was reached within 78 h while the rate of pABA production was highest when ethanol was still available (r_0–46 h_ = 39.52 μmol/g×h, r_46–78 h_ = 7.3 μmol/g×h). Albeit pABA titer was only slightly higher on GLY/ETH than on GLC, productivity was significantly greater (r_GLY/ETH_ = 26.7 μmol/g×h, r_GLC_ = 7.2 μmol/g×h).

Further a dependency of the product spectrum on the carbon-source could be observed. The major by-product was phenylethanol (PEA), which is derived from phenylpyruvate, either as an intermediate of PHE degradation or de novo from prephenate (formation of phenylpyruvate has been observed in vivo even though *ARO7* was knocked out [31]) On glucose PEA titers even exceeded pABA titers. However on GLY/ETH formation of PEA was reduced (threefold) in favour of increased selectivity for pABA. Further on GLY/ETH it was observed that PEA production early in the fermentation correlated directly with the decline of PHE (cf. Additional file 3). Tracer experiments showed that growing the strain solely on U-^13^C glucose led to no significant labelling of PEA (mean enrichment 3 %) at the end of the exponential growth (where PHE was depleted), proving that de novo synthesis had not occurred. In stationary phase, however, PEA appeared to be partially ^13^C enriched (mean enrichment 29 %), indicating de novo synthesis from chorismate (cf. Additional file 3 for details). No other aromatic by- or downstream products of the shikimate pathway were found, in particular no accumulation of aromatic amino acids was observed. The tryptophan degradation product tryptophol was identified in GC–MS samples of the tracer experiments but appeared to be unlabelled and hence originated from tryptophan degradation only (not de novo synthesized). Further dihydrofolate and folate (which pABA is a precursor for) were also not found to be present in the media in detectable concentrations (LOD < 4 µM).

The advantageous stoichiometry for pABA formation on GLY/ETH can explain the initial observation that a major fraction of pABA production on glucose only occurred late in the fermentation after the diauxic-shift: When glucose was consumed ethanol and glycerol were reassimilated, and the major amount of aromatics formed. The fact that production and growth only proceeded until ethanol was depleted indicates that ethanol was essential in order to sustain a viable metabolism of the cell. This, together with the clues from EMA and the tracer experiments, can explain the observed “sweet-spot” of the GLY:ETH ratio for pABA production: Ethanol concentration has to be limited in order to not dominate carbon supply for the cell (due to faster utilization kinetics) but apparently also needs to be high enough to support metabolism and allow sufficient glycerol utilization. The in vivo determined ideal glycerol to ethanol uptake-ratio of 0.5 C-mol/C-mol, which delivered the highest pABA yields, also correlates with the optimum ratio (between 0.5 and 2 C-mol/C-mol) predicted by modelling. Interestingly, further enforcing higher glycerol uptake affected pABA titer negatively and also slightly diminished the yield. This was most likely due to the impaired glycerol uptake mechanism of laboratory yeast [32], presumably resulting in an imbalanced metabolism of the cell if ethanol share in the carbon-source is too low.

**Fig. 7:**
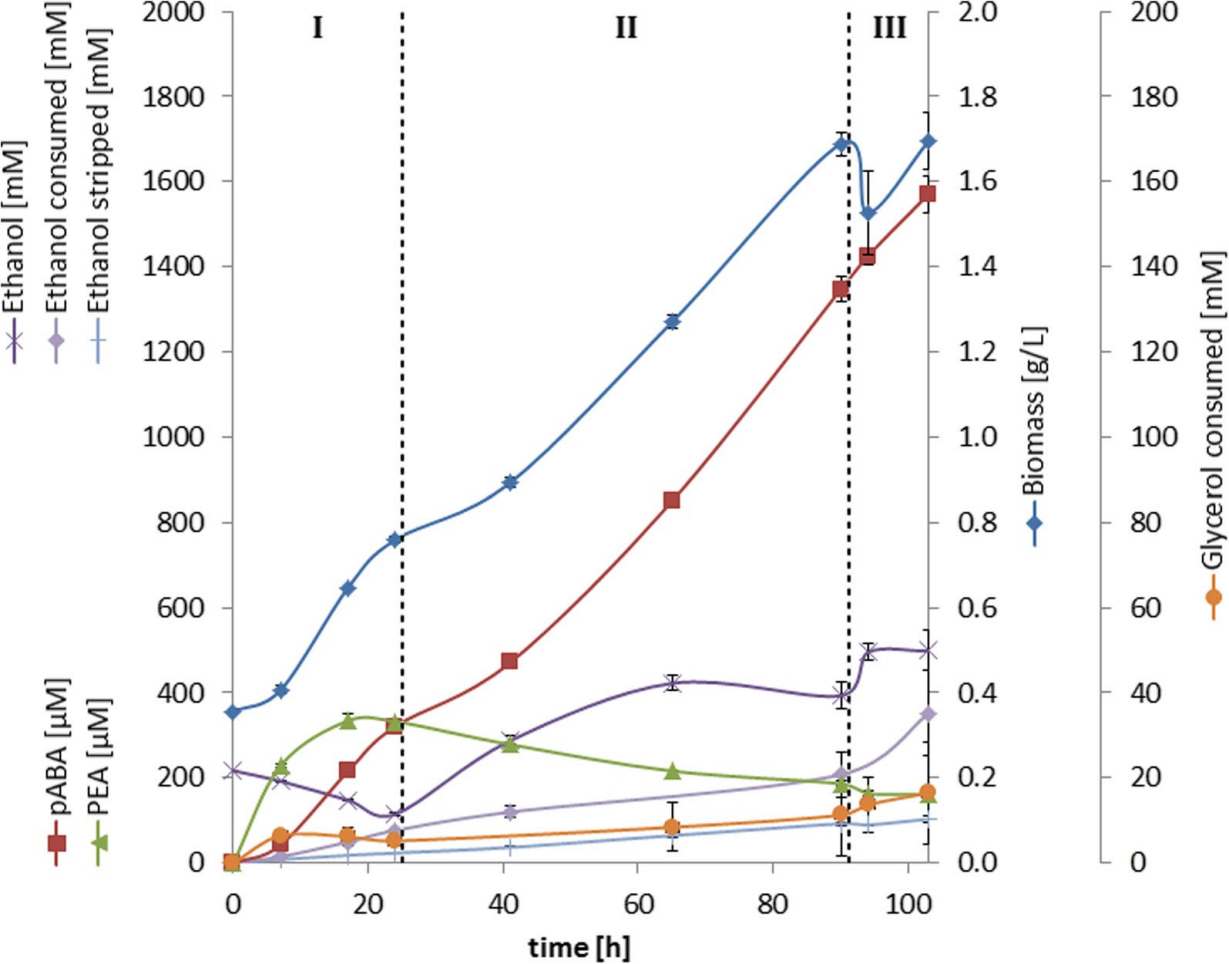
Substrate uptake and aromatic product formation over time in a bioreactor fermentation on GLY/ETH with continuous ETH-feed. Profiles of consumed substrates have been adjusted/corrected for dilution to reflect actual substrate uptake. *Dashed vertical lines* indicate activation/increase of the feed with CDM + ETH respectively: I = batch-phase, II = 6 mL/h, III = 21 mL/h

To maximize pABA production a fed-batch bioreactor experiment with a high starting cell density was conducted. A continuous feed-stream was supposed to counteract stagnation of growth due to degradation of abundant aromatic amino acids and to further improve pABA production and yield. This also intended to explore if higher GLY utilization could be achieved by limiting ETH availability. In theory this would compensate for slower kinetics of GLY uptake and minimize ETH loss by evaporation. Utilization of a greater share of GLY while minimizing ETH usage was also anticipated to benefit product yield (cf. “In vivo restrictions of carbon-source utilization and impact on aromatic product spectrum” section). Using an ETH-feed rate similar to the observed ETH uptake rate (cf. “Strain cultivation**”** section for process details) stripping of ETH was successfully minimized, while upholding GLY utilization (7.4 % consumed) as can be seen from Fig. 7. Notably the highest GLY utilization and also growth was observed towards the end of the fermentation, when ethanol concentration peaked, due to increased feed rate. This allowed the highest pABA titer (1.57 mM) and carbon yield (2.64 %) in this study to be reached (Table 3). Besides that, up to 100 µM pHBA were also detected. Further the PEA titer could be successfully minimized, which benefited the fraction of pABA (>83 % by titer in mM) of total aromatics. The sharply increasing PEA titer in the initial hours largely corresponded to the decline of initial PHE in the medium (data not shown), this correlated with the results from the tracer experiments, where most PEA on GLY/ETH was derived from PHE degradation and little to no PEA being formed de novo. The steady supply of aromatic amino acids in the feed phase enabled the use for growth rather than degradation, as can be seen by a constant drop in the levels of PEA, while no accumulation of amino acids could be observed (data not shown).

**Table 3:** Maximum titers and product carbon yields of key aromatic compounds and total aromatics obtained from batch bioreactor fermentations

Compound	pABA	PEA	Total^a^
Titer (mM)	1.57	0.16	1.9/1.67 de novo
(mg/L)	215	19.5	270
Yield (%)	2.64	0^b^	2.8^b^

Yields are based on dilution/ evaporation corrected concentrations to account for actual substrate utilization

^a^Total aromatics titer is comprised of pABA, PEA, pHBA and tryptophol

^b^Carbon yields of PEA and total aromatics were estimated to reflect only the de novo synthesized fraction

### Carbon-source utilization helps to balance aromatics production

It was recently shown that ethanol stress reduced the production of the aromatic amino acids higher alcohols, including PEA [33]. This was believed to happen via upregulation of the genes *ARO8*, *ARO9* and *ARO10*. However these genes evoke in combination the formation of PEA only via catabolism of aromatic amino acids, which can in the present study not fully explain the observed accumulation of PEA. We recently demonstrated that the conversion of chorismate to prephenate/phenylpyruvate in a ∆*aro7* strain proceeds also spontaneously [31], the reaction rate is merely a matter of substrate and product concentrations, temperature and pH. Assuming that pABA production is kinetically limiting, in a situation of high flux into the shikimate pathway, chorismate could be accumulating, leading to an overflow into PEA. A similar flux overflow resulting in accumulation of PHE and TYR has been reported when producing pABA in *E. coli* [25]. During growth on GLY/ETH overall conversion rates are lower, including the shikimate pathway. Thus the lower flux into the chorismate pool improves balancing with the flux into pABA production and chorismate does not overflow into PEA. Hereby the introduction of the alternative carbon sources helps to balance the pABA synthesis pathways.

Current pABA production appears to be constrained by GLY utilization: If growth is limited (either by means of low ETH concentrations or depletion of aromatic amino acids) then GLY is hardly utilized. If ETH and aromatic amino acids are present in excess, more GLY is utilized, but flux overflow and degradation of aromatic amino acids also diverts carbon to PEA and more ETH is lost via evaporation. The uptake of GLY in *S. cerevisiae* laboratory strains has been described as partially defective and can be improved through over-expression of *STL1*, *GUT1*_*CBS6412*-*13A*_ and *GUT2* [32]. This could help to not only enhance growth but also production on glycerol: It may allow reduction of the ethanol share of the carbon-source, simultaneously reducing process costs and presumably also resulting in further improved yield while utilizing a more sustainable carbon-source.

## Conclusions

The observed titer of 1.6 mM pABA is the highest in *S. cerevisiae* reported to date and the second highest in a biological system. This demonstrates the potential of *S. cerevisiae* as a microbial cell factory for aromatics. It could also be shown that the variability in different *ABZ1* and *ABZ2* sequences of sequenced yeast strains has great impact on pABA production. Further this study demonstrates the potential of metabolic engineering when rationally making use of metabolic modelling and genetic engineering: As predicted by modelling it could be shown that production of pABA from a combined glycerol-ethanol carbon-source was more efficient than from glucose. The benefits are twofold: On the one hand stoichiometry allowed a higher yield (as predicted by modelling); on the other hand kinetic limitations (that otherwise result in flux overflow) were reduced resulting in increased selectivity for the desired compound pABA and reduction of the overflow metabolite PEA. This allowed a more than sixfold increase in product titer and almost eightfold increase in yield over the previous benchmark [9]. In the future enhancing glycerol uptake and metabolization should help to increase the pABA yield and titer.

## Methods

### Metabolic network analysis

Metabolic modelling was conducted by means of elementary mode analysis using EFMTool [34]. The framework was modified from what has been described before [29]. In particular metabolic networks were amended with additional reactions for pABA formation and glycerol/ethanol uptake. The networks are given in Additional file 2.

### Media and strain maintenance

Saccharomyces *cerevisiae* strains were propagated on yeast extract peptone dextrose (YPD) medium, 1 litre was composed of 10 g yeast extract, 20 g peptone and 20 g dextrose (d-glucose) in H_2_O. For strain construction the antibiotics G418 (geneticin) and clonNAT (nourseothricin) were added into the medium to final concentrations of 200 and 100 mg/L, respectively. Also for strain construction as well as maintenance selective synthetic complete medium was used. It was made up from 6.8 g/L yeast nitrogen base without amino acids (Y0626 Sigma) and 1.92 g/L drop-out supplement without uracil (Y1501 Sigma) with 20 g/L glucose or 10 g/L glycerol and 30 g/L ethanol as carbon-source. Solid media contained 20 g/L agar.

For cultivation of yeast chemically defined minimal medium (CDM) was used, 1 l was composed of: 2 g (NH_4_)_2_SO_4_, 1 g (NH_4_)_2_HPO_4_, 5.28 g NaH_2_PO_4_, 0.5 g MgSO_4_·7 H_2_O, 0.015 g EDTA, 1 g KCl, 0.15 g CaCl_2_·2 H_2_O, 0.85 g Na_2_HPO_4_ amended with trace elements and vitamins in aqueous solution. Trace elements (per 1 l H_2_O): 4.5 mg ZnSO_4_·7 H_2_O, 3 mg Fe(III)Cl_3_·6 H_2_O, 1 mg H_3_BO_3_, 0.85 mg MnSO_4_·H_2_O, 0.4 mg NaMoO_4_·2 H_2_O, 0.3 mg CoCl_2_·6 H_2_O, 0.3 mg CuSO_4_·5 H_2_O, 0.1 mg KI. Vitamins (per 1 l): 3 mg *myo*-inositol, 1 mg calcium d-pantothenate, 0.3 mg thiamine hydrochloride, 0.075 mg pyridoxine hydrochloride, 0.0015 mg d-biotin. In case of the knock-out strain 76 mg/L tyrosine, phenylalanine and tryptophan were supplemented, while for the background strain 0.2 mg/L pABA were essential for growth. The pH was adjusted to 6, carbon-sources were glucose (GLC) glycerol (GLY), ethanol (ETH) or a glycerol-ethanol mix (GLY/ETH) with amounts as given in Fig. 4 for the respective experiments. Concentrations of carbon-sources where chosen aiming at comparable amounts of C-mol, e.g. when using 30 g/L ethanol even in case of <50 % substrate loss by evaporation an equivalent amount of C-mol as in the glucose experiment was available over the course of the fermentation. For the tracer experiments 20 g/L fully labelled U-^13^C glucose (Cambridge Isotope Laboratories, Tewksbury, MA) or 2 g/L of fully labelled ^13^C ethanol (99 %, ICON isotopes, Summit, NJ) and 20 g/L unlabelled glycerol were used as carbon-source. Medium for the bioreactor experiment initially contained the twofold ammonium source and 20 g/L glycerol + 10 g/L ethanol. The feed solution was CDM also with the two-fold ammonium source and 10 % ethanol (v/v).

*Escherichia coli* plasmid maintenance strains were cultivated in lysogeny broth (LB) supplemented with 100 mg/L ampicillin. Strains were grown at 30 °C with 200 rpm orbital shaking. All cryo-cultures were stored in 25 % glycerol at −80 °C.

### Strain and plasmid construction

Strain construction was based on the *S. cerevisiae* strain CEN.PK113-5D. *ABZ1* and *ABZ2* genes originated from different sequenced and commercially available wine yeast strains (Table 6). Cloning was routinely conducted following standard protocols [35] and using enzymes obtained from New England Biolabs to manufacturer instructions. In particular Q5^®^ Hot Start High-Fidelity DNA polymerase and high-fidelity (HF^®^) restriction endonucleases were used. For sub-cloning the *E. coli* strain DH5α was utilized. Plasmids and PCR products were purified using the Thermo Scientific GeneJET Plasmid Miniprep and PCR purification kits. Cloned PCR products were verified by sequencing (Sanger service by AGRF, Brisbane, AU).

The *TRP3* deletion was carried out by replacing the target gene via homologous recombination, integrating the natMX antibiotic marker as described in [36] using the lithium acetate method to transform the cells [37]. Successful integrations/deletions were verified by PCR using primers binding inside and outside of the target/marker gene (verA–verD) as outlined in [38]. The same principles were used for *ARO7* deletion/*ARO4*^*K229L*^ integration with a few differences: After incorporating the point mutation in codon 229 of the original *ARO4* (as already described in [8]), which changes the respective amino acid residue from Lys to Leu and renders the protein feedback inhibition resistant, it was cloned onto the plasmid vector pCV3. pCV3-*ARO4*^*K229L*^ was used directly to obtain an integration cassette comprised of the *ARO4*^*K229L*^ gene fused with the *POL4* terminator and the kanMX marker that would integrate at the *ARO7* locus, thus replacing it and putting the *ARO4*^*K229L*^ under control of the *ARO7* promoter, which not only simplified experimental efforts but also resulted in a positive feedback loop for expression. The constructed strain (CEN.PK113-5D Δ*trp3*::natMX Δ*aro*7::kanMX-*ARO4*^*K229L*^, PABA0) served as the host for the pABA expression plasmids.

Plasmid vectors for expression of the genes for pABA formation from chorismate were constructed based on pSP-G1 [39]. The *ABZ1* genes were always cloned to the *TEF1* promotor’s multi-cloning site, while the *ABZ2* genes were cloned into the *PGK1* promotor’s polylinker. The primers used to amplify the respective integration/ deletion cassettes and cloning inserts were purchased from Integrated DNA Technologies, the sequences can be found in Table 4. For cloning, standard desalted primers where used, while for deletion/integration PAGE-purified primers proved to be significantly more efficient.

**Table 4:**
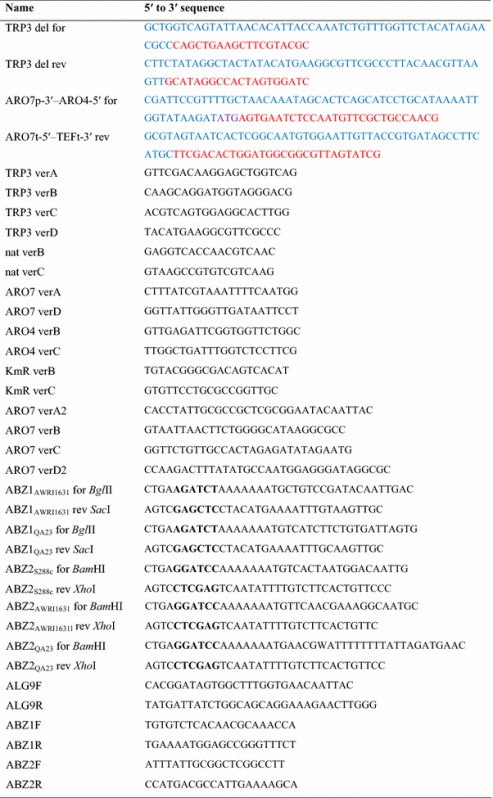
Primers used in this study

Annealing sites in primers for amplification of integration cassettes are shown in red, while overhangs for homologues recombination have been marker blue. For cloning primers the restriction sites are indicated in bold letters

Vectors were constructed in a way that allowed conclusive comparison of the different genes against each other without having to compare each gene separately. Plasmids used in this study are given in Table 5. The plasmids were transformed to the PABA0 strain, genotypes of the final transgenic production strains (CEN.PK113-5D Δ*trp*3::natMX Δ*aro*7::kanMX-*ARO4*^*K229L*^ pSP-G1-*ABZ1*-*ABZ2*, PABA1–PABA5) can be found in Table 6.

**Table 5:** Plasmids used in this study as template for amplification of deletion markers and over-expressing of heterologous genes

Name	Details	Origin
pUG74	PCR template vector, source for loxP-flanked natMX gene deletion marker	[40]
pCV3-*ARO4* ^*K229L*^	*ARO4* ^*K229L*^ expression vector, source for *ARO4* ^*K229L*^–kanMX integration cassette	[8]
pSP-G1	Ura^+^ selectable double expression vector, contains *TEF1*–*PGK1* bidirectional promoter	[39]
pSP-G1-*ABZ1*_*AWRI1631*_-*ABZ2*_*AWRI1631*_½	pSP-G1 with *ABZ1* and *ABZ2* cloned under control of *TEF1* and *PGK1* promoter respectively	This study
pSP-G1-*ABZ1*_*AWRI1631*_-*ABZ2*_*AWRI1631*_ *full*	pSP-G1 with *ABZ1* and *ABZ2* cloned under control of *TEF1* and *PGK1* promoter respectively	This study
pSP-G1-*ABZ1*_*AWRI1631*_-*ABZ2*_*S288c*_	pSP-G1 with *ABZ1* and *ABZ2* cloned under control of *TEF1* and *PGK1* promoter respectively	This study
pSP-G1-*ABZ1*_*AWRI1631*_-*ABZ2*_*QA23*_	pSP-G1 with *ABZ1* and *ABZ2* cloned under control of *TEF1* and *PGK1* promoter respectively	This study
pSP-G1-*ABZ1*_*QA23*_-*ABZ2*_*QA23*_	pSP-G1 with *ABZ1* and *ABZ2* cloned under control of *TEF1* and *PGK1* promoter respectively	This study

**Table 6:** *Saccharomyces cerevisiae* strains used in this study as source of gDNA for production of PCR templates and host organism for genetic engineering

Strain	Genotype/plasmid	Note	Origin
S288c	MATα SUC2 gal2 mal2 mel flo1 flo8-1 hap1 ho bio1 bio6	Reference strain, source of *ABZ2*_*S288c*_ http://www.yeastgenome.org/strain/S288C/overview	[41]
AWRI1631		Wine yeast strain, source of *ABZ1*_*AWRI1631*_, *ABZ2*_*AWRI1631full*_ and *ABZ2*_*AWRI1631*½_ http://www.yeastgenome.org/strain/AWRI1631/overview	[42]
Lalvin QA23		Wine yeast strain, source of *ABZ1*_*QA23*_ and *ABZ2*_*QA23*_ http://www.yeastgenome.org/strain/LalvinQA23/overview	[43]
CEN.PK113-5D	MATa ura3-52 MAL2-8^c^ SUC2	Background strain used to construct the production strains http://www.yeastgenome.org/strain/CENPK/overview	Euroscarf
PABA0	CEN.PK113-5D Δ*trp*3::natMX Δ*aro*7::*ARO4* ^*K229L*^-kanMX pSP-G1	Indole-3-glycerol-phosphate synthase and chorismate mutase knock-out strain with feedback resistant 3-deoxy-D-arabino-heptulosonate-7-phosphate (DAHP) synthase	This study
PABA1	PABA0 pSP-G1-*ABZ1*_*AWRI1631*_-*ABZ2*_*AWRI1631*_½	PABA0 strain with aminodeoxychorismate synthase from AWRI1631 and aminodeoxychorismate lyase from AWRI1631 over-expressed	This study
PABA2	PABA0 pSP-G1-*ABZ1*_*AWRI1631*_-*ABZ2*_*AWRI1631*_ *full*	PABA0 strain with aminodeoxychorismate synthase from AWRI1631 and aminodeoxychorismate lyase from AWRI1631 over-expressed	This study
PABA3	PABA0 pSP-G1-*ABZ1*_*AWRI1631*_-*ABZ2*_*S288c*_	PABA0 strain with aminodeoxychorismate synthase from AWRI1631 and aminodeoxychorismate lyase from S288c over-expressed	This study
PABA4	PABA0 pSP-G1-*ABZ1*_*AWRI1631*_-*ABZ2*_*QA23*_	PABA0 strain with aminodeoxychorismate synthase from AWRI1631 and aminodeoxychorismate lyase from QA23 over-expressed	This study
PABA5	PABA0 pSP-G1-*ABZ1*_*QA23*_-*ABZ2*_*QA23*_	PABA0 strain with aminodeoxychorismate synthase from QA23 and aminodeoxychorismate lyase from QA23 over-expressed	This study

### Strain cultivation

250 mL Erlenmeyer baffled cell culture vent cap polycarbonate flasks (Corning^®^, Corning, NY) were used for shake-flask experiments (culture volume being 10 % of total) using a Multitron 3-stack incubation shaker with 25 mm shaking throw at 200 rpm (INFORS HT, Bottmingen, Schweiz) for incubation of shake-flasks. Experiments were carried out in biological triplicates, single colonies from solid medium were used to inoculate primary pre-cultures on liquid medium (CDM). After overnight growth a secondary pre-culture was inoculated to a defined OD_660_. Cells from log-phase were used to inoculate the main-culture to an OD_660_ of 0.8 in case of *ABZ1*/*ABZ2* performance screening on glucose and 0.2 for all other shake-flask experiments. Growth was monitored by means of OD_660_ and pH, collecting supernatant for analysis every 3 h during exponential growth and twice a day in stationary phase (after 9 h in case of GLC, after 24 h in case of GLY/ETH).

Fed-batch bioreactor experiments were carried out in duplicates using a BIOSTAT^®^ B (Sartorius, Göttingen, Germany). Shake-flask pre-cultures on GLY/ETH were kept exponential (by transferring cells from cultures approaching end log-phase to fresh medium, while adjusting volume to not exceed a final OD of 5 in order to suffice oxygen demand) until enough cells were collected to inoculate 2 × 400 mL medium to an OD of 1.8 or 4.4 for the respective experiments. Cells were washed twice with fresh medium, resuspended in 24 mL thereof and transferred into the bioreactors. Feeding was started when initial ethanol was nearing depletion (inferred by retardation of growth by means of OD). Feed-flow was set to 100 µL/min, corresponding to the approximate ethanol uptake rate (predetermined in shake-flasks), adjusted to the actual OD. In the final stage (cf. Fig. 7) the feed was increased by 3.5-fold. Aeration was kept at a minimum (0.16 L/min) in order to minimize stripping and foaming. However the system was controlled to be aerobic with dissolved oxygen no lower than 80 %. The pH was controlled at 6 using 1 M H_3_PO_4_ and 10 % NH_4_OH. All fermentations were conducted at 30 °C.

### Metabolite analysis

Metabolites were analysed using the same materials and methods as described before [8] to determine extracellular concentrations of aromatics (pABA, anthranilate, pHBA, PHE, TYR, TRP, PEA, tryptophol and folate) as well as glucose, glycerol, ethanol and organic acids. To determination yields accurately, evaporation was respected: For shake-flasks evaporation of total liquid was estimated gravimetrically, while ethanol evaporation was approximated using a predetermined correlation between ethanol concentration and evaporation of ethanol from CDM + GLY/ETH over time to estimate loss of ethanol during fermentation incrementally. The errors of the yields were based on maximum and minimum yields determined with the boundaries for the ethanol evaporation, as this exceeded standard deviation between the triplicates. For the bioreactor experiments the method described in [44] was employed to determine evaporation of water and stripping of ethanol. Yields (in percent C-mol/C-mol) were calculated from the evaporation corrected titers, while only the metabolized fraction of the carbon-source was respected.

^13^C labelled tracer cultivations were performed on fully labelled glucose (d-Glucose–^13^C_6_ 99 atom %; Isotec, Germany) or on a mixture of fully labelled ethanol (^13^C_2_ 99 atom %; Icon Isotopes, USA) and naturally enriched glycerol. Amino acid labellings were obtained from cell hydrolysates and analysed using gas chromatography—mass spectrometry (GC–MS) described previously [9, 31]. For this 0.2 mL of supernatant sample or 0.05 mL of cell hydrolysate were dried under a stream of N_2_ and subsequently derivatized with* N*-methyl-*tert*-butyldimethylsilyl-trifluoroacetamide (MBDSTFA; Macherey–Nagel, Düren, Germany) as described before [31]. For pABA the ion cluster m/z = 308–315 was analysed. It represents the m-57 fragment (full carbon backbone of pABA) of MBDSTFA derivatized pABA. The volatile PEA was extracted into hexane (equal volume to sample, vortex, decant) and directly injected into GC–MS. For PEA, GC–MS settings were modified from the above, by applying a 1:10 split injection. The ion cluster m/z 122-130 contained the full carbon backbone of PEA and was used to calculate enrichment. Generally, labelling distributions were corrected for all naturally occurring stable isotopes in the derivatized molecules that do not belong to the biological carbon backbone using the software IsoCor [45]. The corrected mass distributions were then used in IsoCor to calculate the mean enrichment of compounds using relative summed fractional labelling (SFL) calculated as follows:

$${\text{SFL}} = {{\left( {0 \times {\text{I}}_{{{\text{m}} + 0}} + 1 \times {\text{I}}_{{{\text{m}} + 1}} + 2 \times {\text{I}}_{{{\text{m}} + 2}} \cdots + {\text{n}} \times {\text{I}}_{{{\text{m}} + {\text{n}}}} } \right)} \mathord{\left/ {\vphantom {{\left( {0 \times {\text{I}}_{{{\text{m}} + 0}} + 1 \times {\text{I}}_{{{\text{m}} + 1}} + 2 \times {\text{I}}_{{{\text{m}} + 2}} \cdots + {\text{n}} \times {\text{I}}_{{{\text{m}} + {\text{n}}}} } \right)} {\text{n}}}} \right. \kern-0pt} {\text{n}}}$$

where *I* is the intensity of the (*m* + *i*) ion in the ion cluster and *n* the number of carbon atoms in the analysed mass fragment.

### Quantitative real-time PCR

To determine the relative mRNA levels of the different over-expressed *ABZ1* and *ABZ2* genes, PABA strains were cultivated in shake-flask experiments on CDM + GLC, as described earlier. Samples (1–2 mL) from exponential phase (OD_660_ = 1) were centrifuged to pellet cells; cell pellets were resuspended in 1 mL TRIzol^®^ (Ambion) and stored at −80 °C until extraction (no longer than 10 days). RNA was extracted using the PureLink^®^ RNA Mini Kit (Ambion) in combination with RQ1 RNase-Free DNase (Promega) for on column DNAse treatment. mRNA (100–1000 ng) was reverse transcribed using Oligo d(T)_18_ primers and SuperScript^®^ III reverse transcriptase (Invitrogen) in combination with RNase Inhibitor, Murine (NEB) according to the manufacturer’s instructions. qPCR reactions were carried out in a CFX96 Touch™ Real-Time PCR Detection System, using the SsoAdvanced™ Universal SYBR^®^ Green Supermix (Bio-Rad) outgoing from 10 to 100 ng RNA equivalent. Primers for mRNAs were unified to amplify regions homologous among the different *ABZ1* and *ABZ2* genes respectively, the sequences are listed in Table 4. Relative mRNA levels were determined by normalising to the housekeeping gene *ALG9* [46] using the ΔCq method for each replicate individually as done before [8]. Relative expression levels were averaged for each individual gene among the different strains and two different sampling points in exponential phase. Error is standard deviation thereof.

## Additional files

10.1186/s12934-016-0485-8 Sequence analysis & discussion of *ABZ1* & *ABZ2*. XLS-file containing sequence alignments & codon usage analysis of the different examined ABZ1 and ABZ2 genes.
10.1186/s12934-016-0485-8 EMA networks & EFM plots for pABA production from different carbon-sources. XLS-file containing stoichiometric models for elementary mode analysis & result figures of product vs. biomass yield plots.
10.1186/s12934-016-0485-8 Design & evaluation of tracer experiments. PDF-document with additional information regarding results and discussion of the 13C enrichment experiments.

## Abbreviations

AAA: aromatic amino acids
CDM: chemically defined medium
DAHP: 3-deoxy-d-arabino-heptulosonate-7-phosphate
DHS: 3-dehydroshikimate
DHQ: 3-dehydroquinate
E4P: erythrose-4-phosphate
EMA: elementary mode analysis
ETH: ethanol
GLC: glucose
GLY: glycerol
LOD: level of detection
OD_660_: optical density at 660 nm
ORF: open reading frame
pABA: *para*-aminobenzoic acid
PEA: phenyl ethanol
PEP: phosphoenolpyruvate
PET: polyethylene terephthalate
pHBA: *para*-hydroxybenzoic acid
PHE: phenylalanine
PPY: phenyl pyruvate
S3P: shikimate-3-phosphate
SFL: summed fractional labelling
TRP: tryptophan
TYR: tyrosine
