# Supplementary material for: Production of para-aminobenzoic acid from different carbon-sources in engineered Saccharomyces cerevisiae
Source: Microb Cell Fact. 2016 May 26;15:89. doi: 10.1186/s12934-016-0485-8 (PMC4882779; doi:10.1186/s12934-016-0485-8)
Supplement: Supplementary file 3 — 10.1186/s12934-016-0485-8 Design & evaluation of tracer experiments. PDF-document with additional information regarding results and discussion of the 13C enrichment experiments. [file 12934_2016_485_MOESM3_ESM.pdf]

Two objectives were pursued with the tracer experiments, described in the following

1. The highest pABA carbon yields could be achieved on medium high in glycerol (figure S1). To identify to what degree glycerol contributes to pABA production the fraction of each carbon source incorporated in pABA was determined. This was done in a tracer experiment with natural (^{12}C) glycerol and fully (^{13}C) labelled ethanol, used in a 20:2 ratio (by weight). To allow comparability to the experiments with different carbon source composition the uptake ratios for other GLY:ETH concentrations were calculated (table S1). Based on this the validity of the results from the enrichment experiment (table S2) can be assessed for the other ratios.

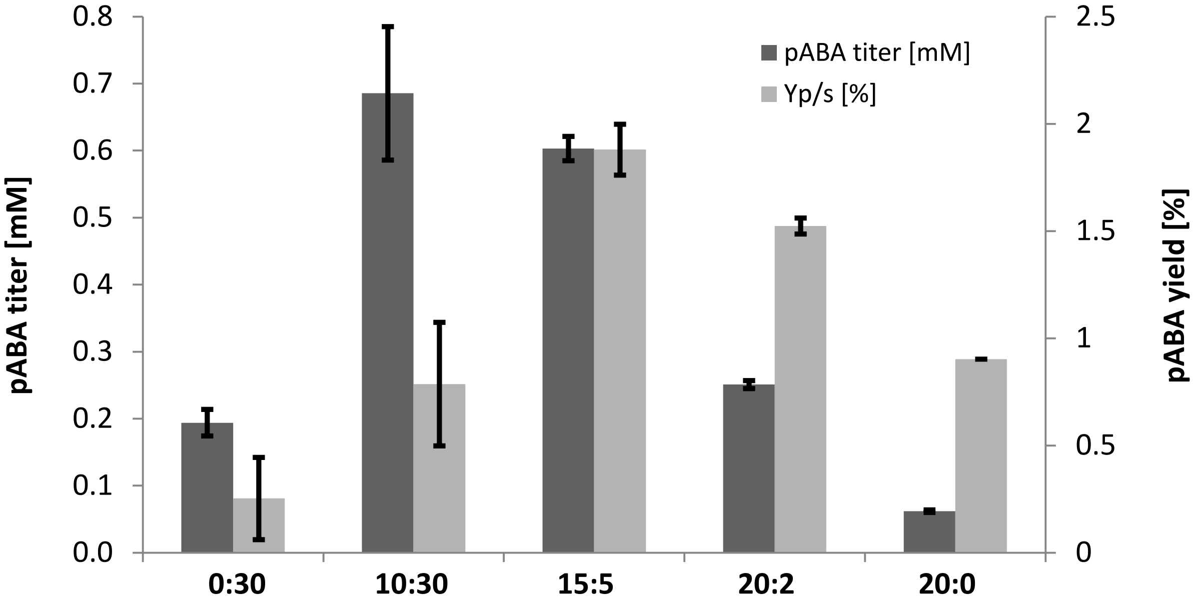

Figure S1: Titers and yield obtained from PABA4 grown on different GLY:ETH ratios. pABA titers are evaporation corrected, $Y_{p/s}$ = carbon yield of product to substrate.

At a 15:5 [g/g] ratio of GLY:ETH in the medium the highest product yield and titer (c.f. figure S1) were possible. When further decreasing the ratio, a threshold was reached where glycerol utilization and product yield dropped sharply. This gives rise to the hypothesis that a synergistic effect is present, rather than a separated co-metabolism. The non-linear correlation between employed and utilized carbon at the different ratios (table S1) supports this. Therefore the validity of the estimated contribution of glycerol to pABA production (table S3) has to be considered with caution, especially for the lower GLY:ETH ratios.

Table S1: Ratios of utilized glycerol to utilized ethanol for different initial carbon source compositions

Ratio of initial glycerol to ethanol [g:g]	10:30	15:5	30:10	20:2
Ratio of utilized glycerol to ethanol [mol/mol]	0.00	0.34	0.4	0.74

2. In the course of the fermentations on GLY/ETH it was observed that the decline of the initial PHE concentration during the exponential phase directly correlated with the production of PEA (figure S2). However labelling analysis of PEA from this experiment could not unequivocal prove that PEA formation did not occur *de novo* (from glycerol). Therefore a second tracer experiment on 100% $U^{13}C$ labelled GLC was performed. This allowed unambiguous determination of the origin of the PEA at different stages of the fermentation (table S2).

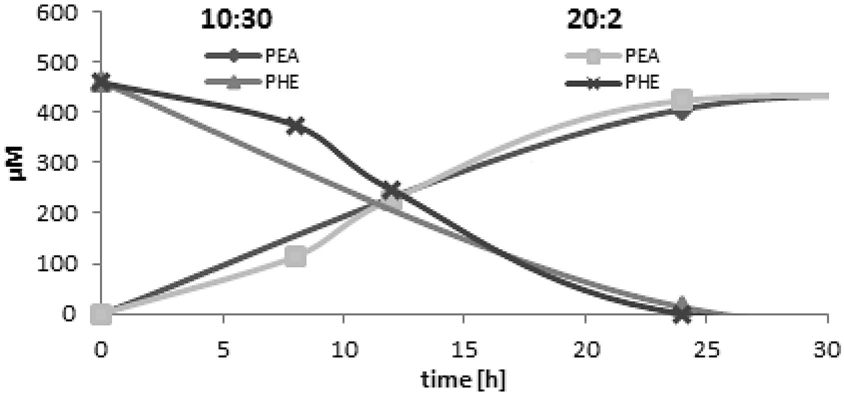

Figure S2: Concentration profile of phenylalanine (PHE) and phenethyl alcohol (PEA) during the initial phase of fermentations on GLY+ETH 20:2 g/L and 10:30 g/L

The results of the GC-MS analysis of the ^{13}C enrichment experiment are collected in table S2. The different labelling of pABA and PEA at two time points in the fermentation is graphically shown in figure S3 by means of the presence of the different isotopomers.

Table S2: Percentage ^{13}C content of total carbon (mean enrichment) in pABA and PEA at different stages

$U^{12}C$-GLY:$U^{13}C$-ETH (20:2 g/L)		pABA	PEA
$\%^{13}C$	end-log (24 h)	64±0.3	1±0.5
	end-stationary (48 h)	69±0.5	n.d.

CDM $U^{13}C$-GLC (20 g/L)		pABA	PEA
$\%^{13}C$	end-log (13 h)	n.d.	2±0.6
	start-stationary (24 h)	n.d.	3±0.7
	end-stationary (48 h)	97±0.	29±0.9

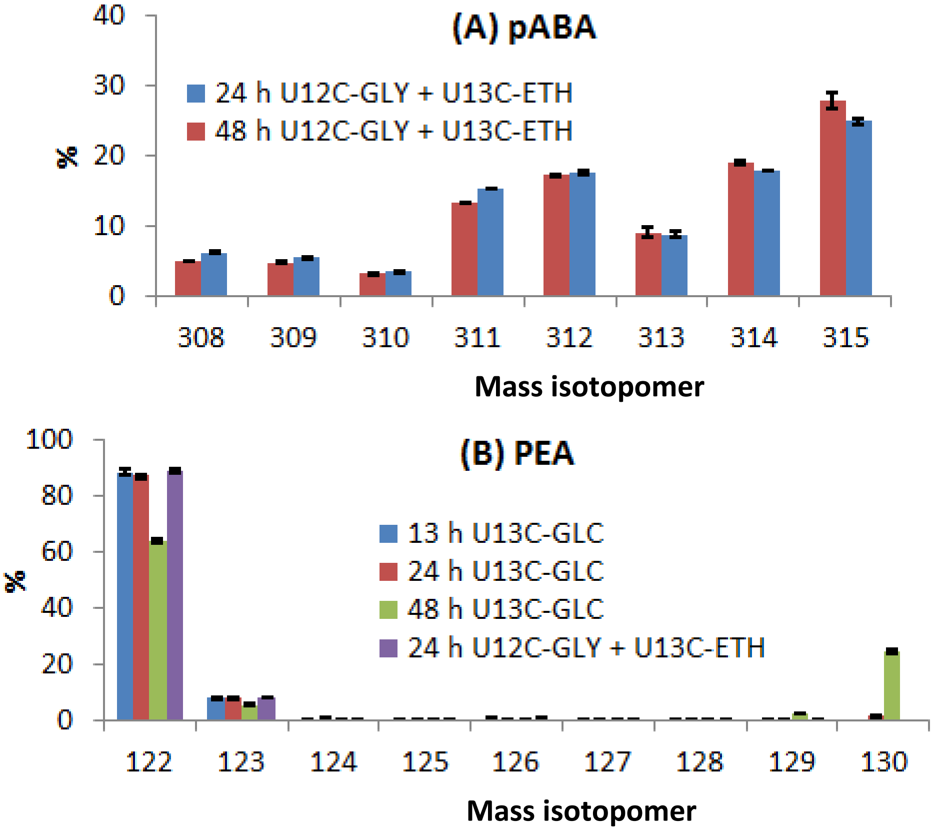

Figure S3: Distribution of the isotopomers of (A) pABA and (B) PEA with different masses obtained from 20 g/L U^{12}C-glycerol + 2 g/L U^{13}C-ethanol and 20 g/L U^{13}C-glucose at the different time points

To put the contribution of glycerol/ethanol to pABA in perspective with the overall utilization of carbon the mean enrichment of pABA from the mass isotopomers was determined. Based on this the approx. contribution of glycerol to pABA for other feed-compositions was estimated (table S3).

Table S3: Contribution of glycerol to pABA for different GLY:ETH ratios. Italic values are based on the results from the 20:2 ^{13}C experiment, estimated for the other GLY:ETH uptake ratios (by rule of three).

Ratio of initial glycerol to ethanol [g:g]	10:30	15:5	30:10	20:2
Ratio of utilized glycerol to ethanol [C-mol/C-mol]	0.003	0.52	0.60	1.10
Ratio of carbon derived from glycerol to carbon derived from ethanol incorporated in pABA [C-mol/C-mol]	*0.001*	*0.22*	*0.26*	0.47

1. With a total share of 35% pABA derived from glycerol after 24 h and 31% final it could be shown that glycerol contributes significantly to pABA production. However contribution of glycerol to pABA production is contrary to overall usage in two ways: After 24 h glycerol usage was only marginal, while at the end of the fermentation, where some glycerol was consumed, the contribution to pABA production dropped. This is even more surprising as ethanol was depleted already after 30 h, while pABA was still produced until the end of the fermentation (48 h). Further the ratio of non-labelled to labelled carbon (which corresponds to the ratio of carbon derived from glycerol to carbon derived from ethanol) was lower for pABA than the overall ratio of consumed carbon (cf. table S3).
   It was anticipated that most E4P would be glycerol derived while PEP would mostly be formed from ETH. However the broad distribution of different mass isotopomers (figure 3A) shows that carbon undergoes different interconversions until it reaches shikimate pathway. Labelling of more than the three PEP derived carbons in pABA (i.e. > 43%^{13}C, in fact even the fully labelled isotopomer was abundant) is only possible if also E4P was already labelled. This means that a significant share of carbon flux entering pentose phosphate pathway was derived from ETH.
   The key function of glycerol in the co-metabolism remains uncertain. It could be that it shifts redox balance and as such serves as an "enhancer" helping to elevate product carbon yield by means of improved energy metabolism.

2. The results from the tracer experiment on U^{13}C-GLC show unequivocally that PEA is not produced *de novo* until late in the fermentation, where a fully ^{13}C labelled isotopomer of PEA (m = 130) can be identified. The presence of the 123 isotopomer throughout the fermentation can be explained with the natural isotope distribution of carbon. Therefore it can be assume that 88 – 92% of the PEA formed in the early fermentation (≙ 407 – 424 µM PEA for 460 µM initial PHE) are not a product of anabolic metabolism.
